# A Kidney-Targeted Nanoparticle to Augment Renal Lymphatic Density Decreases Blood Pressure in Hypertensive Mice

**DOI:** 10.3390/pharmaceutics14010084

**Published:** 2021-12-30

**Authors:** Bethany L. Goodlett, Chang Sun Kang, Eunsoo Yoo, Shobana Navaneethabalakrishnan, Dakshnapriya Balasubbramanian, Sydney E. Love, Braden M. Sims, Daniela L. Avilez, Winter Tate, Delilah R. Chavez, Gaurav Baranwal, Mary B. Nabity, Joseph M. Rutkowski, Dongin Kim, Brett M. Mitchell

**Affiliations:** 1 Department of Medical Physiology, College of Medicine, Texas A&M University, Bryan, TX 77807, USA; bethany.goodlett@tamu.edu (B.L.G.); navshobi27@exchange.tamu.edu (S.N.); Dakshna.Bala@childrens.harvard.edu (D.B.); sydney.love99@tamu.edu (S.E.L.); bradensims@tamu.edu (B.M.S.); danielalaavilez@tamu.edu (D.L.A.); winterwinter97@tamu.edu (W.T.); delilahrchavez@tamu.edu (D.R.C.); gauravbaranwal@tamu.edu (G.B.); rutkowski@tamu.edu (J.M.R.); 2 Department of Pharmaceutical Sciences, College of Pharmacy, Texas A&M University, College Station, TX 77843, USA; chang-kang@ouhsc.edu (C.S.K.); eyoo@ncat.edu (E.Y.); Dongin-Kim@ouhsc.edu (D.K.); 3 Department of Veterinary Pathobiology, College of Veterinary Medicine & Biomedical Science, Texas A&M University, College Station, TX 77843, USA; mnabity@cvm.tamu.edu

**Keywords:** kidney, lymphatics, inflammation, immunity, hypertension

## Abstract

Chronic interstitial inflammation and renal infiltration of activated immune cells play an integral role in hypertension. Lymphatics regulate inflammation through clearance of immune cells and excess interstitial fluid. Previously, we demonstrated increasing renal lymphangiogenesis prevents hypertension in mice. We hypothesized that targeted nanoparticle delivery of vascular endothelial growth factor-C (VEGF-C) to the kidney would induce renal lymphangiogenesis, lowering blood pressure in hypertensive mice. A kidney-targeting nanoparticle was loaded with a VEGF receptor-3-specific form of VEGF-C and injected into mice with angiotensin II-induced hypertension or LNAME-induced hypertension every 3 days. Nanoparticle-treated mice exhibited increased renal lymphatic vessel density and width compared to hypertensive mice injected with VEGF-C alone. Nanoparticle-treated mice exhibited decreased systolic blood pressure, decreased pro-inflammatory renal immune cells, and increased urinary fractional excretion of sodium. Our findings demonstrate that pharmacologically expanding renal lymphatics decreases blood pressure and is associated with favorable alterations in renal immune cells and increased sodium excretion.

## 1. Introduction

Almost 1 in 2 U.S. adults have hypertension, and it is uncontrolled in many patients [1]. Despite improved education, public health efforts, and prescribed medications, about 30–60% of hypertensive patients cannot achieve an ideal blood pressure. This is unfortunate as a decrease in systolic blood pressure of even 10 mmHg significantly reduces health risks and improves outcomes [2]. Additional treatment options are needed to help this hypertensive population reduce their blood pressure, ideally into the normotensive range.

Renal immune cell infiltration, inflammation, and sodium retention are strongly associated with hypertension [3,4,5]. Lymphatic vessels transport fluid, electrolytes, cells, and proteins out of the interstitial space to the draining lymph node and then back to the circulation [6,7]. Inflammation-associated lymphangiogenesis is observed upon acute kidney injury and in chronic inflammatory diseases such as diabetic nephropathy and renal carcinoma [8]. In an inflammatory state, an increase in lymphatics tries to help with the removal of immune cells, proteins, and fluid from the organ. We have reported previously that lymphangiogenesis also occurs in the kidney as a compensatory response in various models of hypertension [9,10,11]. However, this compensatory increase in renal lymphatics was not sufficient to resolve the inflammation and hypertension. Genetically inducing renal-specific lymphangiogenesis in mice prevented the development of salt-sensitive hypertension, nitric oxide inhibition (L-NAME)-induced hypertension (LHTN), and angiotensin II-induced hypertension (A2HTN), and this was associated with a reduction in renal immune cell accumulation and increased sodium excretion [9,11,12]. Additionally, genetic induction of renal-specific lymphangiogenesis in mice attenuated existing LHTN, and this was accompanied by an increase in sodium excretion [12].

Here, we wanted to develop a potential translational therapeutic that would specifically target the kidney and promote lymphangiogenesis to decrease blood pressure under hypertensive conditions. We utilized nanoparticle technology for lymphatic-specific growth factor delivery based on previous reports on kidney-targeting nanoparticles [13,14,15]. Other drug delivery systems utilizing nanoparticles exist, and all are intended to improve efficiency, specificity, and bioavailability. Nanoparticles can be designed to exhibit unique physiochemical properties such as size (50~200 nm), shape, and surface chemistry and they can be equipped to carry one or more diagnostic and/or therapeutic agents, release them via an external trigger such as temperature, pH, and enzymes. Also, encapsulation of those therapeutic or diagnostic agents within nanoparticles allows them to enhance their systemic half-lives, and thus, they can be significantly localized to the targeted area. Currently, there are various nanoparticles including micelles, liposomes, nanospheres, nanotubes, nanocapsules, cubosomes, and hydrogels based on their morphologies. Here we tested both micelle and liposome nanoparticles to determine which was more effective.

Our hypothesis was that a kidney-targeted nanoparticle delivering a pro-lymphangiogenic growth factor, a vascular endothelial growth factor receptor-3 (VEGFR-3)-specific isoform of vascular endothelial growth factor C (VEGF-C), would increase renal lymphatic density and decrease blood pressure in male and female mice with A2HTN or LHTN. We also hypothesized that the decrease in blood pressure would be associated with a decrease in pro-inflammatory renal immune cells, an increase in anti-inflammatory renal immune cells, and increased sodium excretion. Due to the long duration to induce salt-sensitive hypertension and the limited number of tail vein injections in mice, this model was not utilized in the current study.

## 2. Materials and Methods

### 2.1. Nanoparticle Development and Characterization

The PLGA-PEG-folate block copolymer was purchased from PolySciTech (West Lafayette, IN, USA), and the Certificate of Analysis exhibiting the NMR, FTIR analysis, and GPC can be seen in Figure S1. Using 50 mg of PLGA-PEG-folate block copolymer, 10 mg of Coumarin 6 dye, 10 mg of FITC-ovalbumin, or 1 mg of VEGF-C was encapsulated into the nanoparticles using a dialysis method to make micelle nanoparticle 1 (NP1). Specifically, the drug and polymer were dissolved in dimethyl sulfoxide (DMSO), and the solution was transferred into a dialysis membrane (MWCO 100,000). The dialysis was carried out for 24 h against DI water. After that, the aqueous particle solution was centrifuged and sonicated to concentrate the particles. To make the liposomal nanoparticle, we used several lipid components including DSPC (120 mg), cholesterol (16 mg), DSPE-PEG-folate (24 mg), and Coumarin 6 dye (10 mg) or FITC-ovalbumin (10 mg). First, all of the above components were dissolved into DCM (10 mL) and then DCM was removed through air flow to make a thin film on the surface of glass vials. Next, 10 mL of PBS was added into the thin film to make liposomal nanoparticle 2 (NP2). The size of the nanoparticles was determined by dynamic light scattering (DLS) using a Zetasizer (Malvern Panalytical, Malvern, UK). The sample concentration was maintained at 0.5 mg/mL. The amount of Coumarin 6 dye encapsulation was derived from its absorbance measurement by dissolving 100 µL of nanoparticles into 900 µL DMSO, which released Coumarin 6 into the DMSO solution. Absorbance was then measured at 444 nm. Using a pre-measured calibration curve of Coumarin 6 absorbance according to its titrated concentration, the encapsulated Coumarin 6 concentration was calculated (NP1: 5 ± 0.35 mg and NP2: 4 ± 0.89 mg for Coumarin 6). Using a pre-measured calibration curve of FITC-ovalbumin absorbance according to its titrated concentration, the encapsulated FITC-ovalbumin concentration was calculated (4 ± 0.51 mg). Encapsulation amount of VEGF-C within NP was determined by ELISA and was ~500 ± 17 µg. All NPs were coated with folates that target them to the proximal tubules of the kidney, given the high expression of folate receptors. A schematic showing the NP assembly can be seen in Figure S2.

The release profiles of Coumarin 6 or FITC-ovalbumin were studied using a membrane tube dialysis method. One mL of nanoparticle solution was added to a dialysis tube (Cutoff MW 3500) and then immersed in 10 mL of PBS containing 0.5% v/v Tween 80 solution. At the given time, the media was collected and replaced. The fluorescence intensity of collected samples was measured at Ex/Em = 444/500 nm (Coumarin 6) or Ex/Em = 488/530 nm for FITC-ovalbumin using a microplate reader. The cumulative release % of both Coumarin 6 and FITC-ovalbumin from the folate nanoparticles can be seen in Figure S3.

For the transmission electron microscopy (TEM, JEOL JEM-2010, Japan) study, sample suspensions were dropped onto a carbon-coated copper grid and dried under the vacuum condition. The grid was then observed under a JEOL transmission electron microscope at an accelerating voltage of 200 kV. To determine in vivo biodistribution, 100 µL of each NP1 (10 mg/mL) or NP2 (10 mg/mL) was injected into female C57BL/6J mice (n = 3) through the tail vein. The fluorescence signals of Coumarin 6 were recorded at Ex/Em = 444/500 nm. Twelve hours after injection, the animals were euthanized, and each organ was collected. The harvested organs were scanned under an In Vivo FX Pro scanner to quantify the NP distribution. All procedures performed in mice were approved by the Texas A&M University IACUC in accordance with the NIH Guide for the Care and Use of Laboratory Animals.

### 2.2. Mice

Wild-type C57BL/6J mice were purchased from Jackson Laboratories (Bar Harbor, ME, USA). Male and female mice 10–14 weeks of age were surgically implanted under inhaled isoflurane (1.5–5%) anesthesia with osmotic mini-pumps (Alzet, model 1004, Cupertino, CA, USA) containing angiotensin II (490 ng/kg/min; BACHEM, Torrance, CA, USA) (A2HTN), followed by topical lidocaine. Another group of male and female mice were given L-NAME (Sigma, St. Louis, MO, USA) in their drinking water (0.5 mg/mL) throughout the duration of the experiment (LHTN). After 2 days, hypertension was confirmed via tail cuff blood pressure in all groups. The mice were then randomized and injected in the tail vein with either a micellar nanoparticle loaded with the VEGFR-3-specific mutant of VEGF-C (VEGF-C c156s) [16] or free VEGF-C protein alone every 3 days over the course of 16 days (4 total tail vein injections). This injection schedule was modeled after previous work [17] and allowed adequate time for vascular remodeling to occur at a level sufficient to impact blood pressure. Additionally, the 3-day rest period extended the life of the fragile murine tail veins while keeping the pro-lymphangiogenic signal consistent. Even with the recovery period, tail veins could only handle 5 injections before they sustained irrevocable damage. All mice were sacrificed 16 days following the initial injection. Mice were euthanized by tissue perfusion under deep isoflurane anesthesia and then cervical dislocation prior to tissue collection. All procedures performed in mice were approved by the Texas A&M University IACUC in accordance with the NIH Guide for the Care and Use of Laboratory Animals.

### 2.3. Blood Pressure

Systolic blood pressure (SBP) readings were taken at various time points via tail cuff. The IITC Life Science noninvasive blood pressure acquisition system for mice (IITC Inc., Woodland Hills, CA, USA) was used for all measurements. Blood pressures were taken after a 30-min acclimatization period in a designated quiet area. During this time, the warming chamber and restraint chambers were allowed to heat to 34 °C. Mice were gently loaded into appropriately sized restraint chambers and left to acclimatize in the warming chamber for 5 min prior to SBP measurements. SBP measurements were derived from the pressure tracings by two independent, blinded investigators.

### 2.4. Serum and Urine Collection and Measures

Serum and Urine Collection and Measures: Mice were placed in metabolic cages (Hatteras, Cary, NC, USA) and allowed to acclimate overnight. Following acclimation, urine was collected for 24 h, concluding with euthanasia. The collected urine was measured. Blood was collected at euthanization via the left ventricle and serum was isolated for later measures. Serum and urine were analyzed by capillary electrophoresis for sodium using a DxC 700 AU Chemistry Analyzer (Beckman Coulter, Brea, CA, USA) and by direct potentiometry for creatinine using a P/ACE MDQ Plus Capillary Electrophoresis System (Sciex, Redwood City, CA, USA). These measures were used to calculate urinary excretion, fractional excretion of sodium (FENa), and creatinine clearance.

### 2.5. Immunofluorescence

At euthanization, kidneys were collected, cut sagittally into halves, and fixed in 10% buffered formalin solution (Sigma, St. Louis, MO, USA) for 48 h. Following fixation, kidney halves were washed in 100% ethanol and embedded in paraffin. Kidney halves were cut into 5–7 μm sections, which were deparaffinized, rehydrated, and permeabilized with 0.1% Triton solution (BioRad, Hercules, CA, USA). Tissue was blocked with 10% AquaBlock solution (EastCoastBio, North Berwick, ME, USA) before being immunolabeled with the lymphatic endothelial cell markers LYVE-1 or podoplanin (goat polyclonal; R&D Systems, Minneapolis, MN, USA) by overnight incubation at 4 °C. Alexafluor 488 or 594 (Life Technologies, Carlsbad, CA, USA) secondary antibodies were used to visualize lymphatic vessels. Samples were incubated with appropriately conjugated secondary antibodies for an hour at room temperature. Negative controls were incubated with only a secondary antibody. All labeled slides were mounted with ProLong Gold anti-fade reagent containing DAPI (Invitrogen, Carlsbad, CA, USA). An Olympus BX51 fluorescence microscopy system with Olympus Q5 camera was used for imaging. Images were captured at 40× magnification using Olympus CellSens imaging software (Olympus, Shinjuku, Tokyo, Japan). The same methods were utilized to image lymphatics in the heart, liver, lung, and skin (10× or 20× magnification).

### 2.6. Renal Immunofluorescence Quantification

From each kidney section, 5–7 images were collected at 20× from pre-determined areas, generally avoiding tissue defects, minor calyx, and large numbers of glomeruli. ImageJ was used to collect area values measuring the total number of podoplanin+ pixels after the threshold was set for positive endothelium.

### 2.7. Maximal Renal Lymphatic Vessel Width Quantification

From each kidney section, images were collected of all artery-associated lymphatic vessels within the cortex at 20×. Lymphatic vessels were identified by the presence of podoplanin staining. ImageJ was used to collect the length in pixels of the widest point of all lumen-containing lymphatic vessels.

### 2.8. Flow Cytometry

At euthanization, kidneys were collected, and the capsules were removed. The tissue was thoroughly minced and digested in buffer containing Collagenase D (2.5 mg/mL, Roche Sigma, St. Louis, MO, USA) and Dispase II (1 mg/mL, Sigma) at 37 °C for 1 h. Digested samples were filtered and rinsed through 100 and 40 μm strainers. The flow-through was centrifuged to isolate cellular components, and red blood cells were lysed in NH4Cl/EDTA. Splenocytes were isolated similarly for use as antibody controls and for forward versus side scatter gating of immune cell size and shape. Renal cells were resuspended in 0.1% FBS solution. To prevent nonspecific Fc binding, samples were incubated with an anti-mouse CD16/CD32 antibody (BD Pharmingen, San Jose, CA, USA) for 10 min on ice. Cells were then incubated with fluorescent-conjugated antibodies against CD45, F4/80, CD11c, and CD3e and filtered through another sterile 40 μm strainer. All antibodies were purchased from BD Biosciences. See Online Table S1 for the descriptive panel. A BD LSR Fortessa X-20 flow cytometer with FACS DIVA software (BD Biosciences, San Jose, CA, USA) was used for acquisition. Populations of up to 500,000 cells were analyzed using FlowJo v10.6.2.1 (FlowJo, LLC, Ashland, OR, USA). CD3e+ populations were quantified within the CD45+ gate. CD3e PacBlue+ populations were negative for fluorophores PE and PerCP-Cy5.5. F4/80+ and CD11c+ populations were quantified within the CD45+/CD3e- gate (Figure S4). These populations were negative for PacBlue, APC, and APC-Cy7 fluorophores. Results are expressed as a percentage of total CD45+ cells per kidney. The gating strategy can be found in Figure S4. Antibody information is found in Table S1.

### 2.9. Statistics

Results are presented as dot plots and means or mean ± SEM. Differences between 2 groups were assessed by a 2-tailed unpaired Student’s *t*-test and between 3 or more groups with a one-way ANOVA followed by the Student–Newman–Keuls post hoc test. The significance level was set at 0.05 for all comparisons. All statistical analyses were performed using SigmaPlot 10 software (Systat, San Jose, CA, USA).

## 3. Results

**Figure 1:**
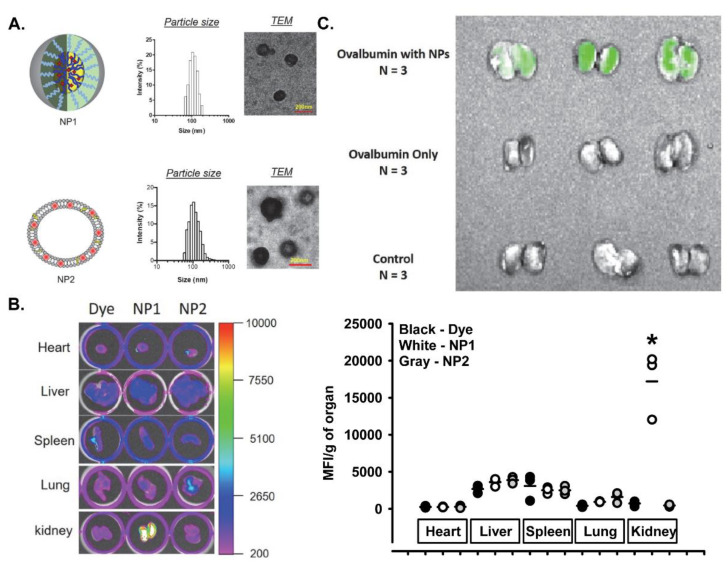
Characteristics of the nanoparticle and ability to target the kidney with dye and fluorescent protein. (**A**) Micellar and Liposome nanoparticles and their size. Scale bar = 200 nm. (**B**) Organs from mice 12 h after a single i.v. injection of Coumarin 6 dye alone, NP1 containing Coumarin 6 dye, or NP2 containing Coumarin 6 dye. Organs were imaged using an In Vivo FX Pro, and the mean fluorescence index (MFI) was calculated and expressed per gram of organ. (**C**) Kidneys from mice 12 h after a single i.v. injection of NP1 containing FITC-Ovalbumin, FITC-Ovalbumin alone, or vehicle alone. Organs were imaged using an In Vivo FX Pro. Results are expressed as dot plots and mean (n = 3 per group) and statistical analyses were performed with a one-way ANOVA followed by the Student–Newman–Keuls post hoc test. * *p* < 0.05 vs. Coumarin 6 dye alone group in the same organ.

We developed two different kidney-targeting nanoparticles, one a micelle (NP1) and the other a liposome (NP2), with an average diameter of 100 nm and containing Coumarin 6 dye for tracking (Figure 1A). The nanoparticles were coated with folates that make them target the proximal tubule cells of the kidney, given their high expression of folate receptors [18,19,20]. The nanoparticles were injected into the tail vein of female C57Bl/6J mice, and 12 h later the mice were euthanized, and the organs collected and imaged for fluorescence. While there was little Coumarin 6 dye in the hearts, livers, spleens, lungs, and kidneys in mice receiving free dye or the liposome nanoparticle (NP2), there was a significant amount of fluorescence specifically in the kidneys of mice injected with NP1, the micelle nanoparticle (Figure 1B).

Next, we determined whether the micelle nanoparticle (referred to as NP from here forward) could deliver protein to the kidneys. Female mice received tail vein injections of NPs loaded with FITC-ovalbumin, free FITC-ovalbumin protein, or vehicle control and were euthanized 12 h later. There was a significant amount of FITC-ovalbumin identified only in the kidneys from mice injected with the NPs containing FITC-ovalbumin demonstrating high specificity of renal protein delivery (Figure 1C).

**Figure 2:**
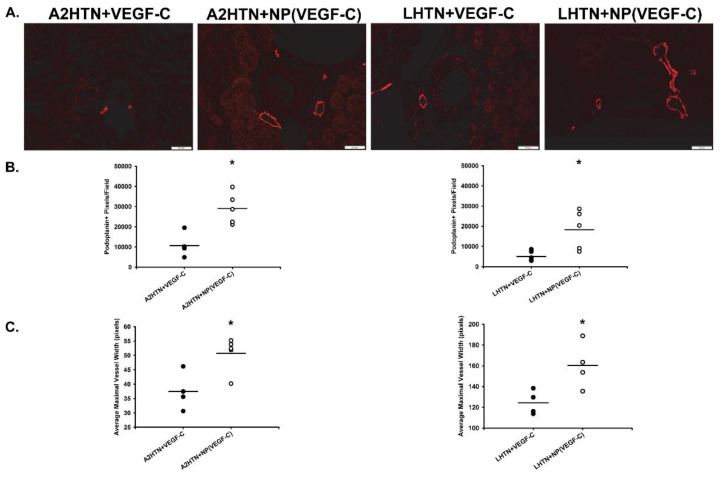
Increased renal lymphatic vessel density in hypertensive mice treated with VEGF-C in nanoparticles. (**A**) Podoplanin immunofluorescence on kidney sections from male and female mice with angiotensin II-induced hypertension (A2HTN) or L-NAME-induced hypertension (LHTN). A2HTN and LHTN mice were injected with VEGF-C in micellar nanoparticles (NP) or VEGF-C alone every 3 days. Scale bars = 50 μm. (**B**) Renal interlobular lymphatic density as determined by podoplanin+ pixel density using ImageJ. Results are expressed as mean (n = 5 mice per group). (**C**) Maximal renal lymphatic vessel width, measured in lumen-containing lymphatic vessels within the cortex. Lymphatic vessels were identified by podoplanin staining and measurements were taken using ImageJ. Results are expressed as mean (n = 4 mice per group). All statistical analyses were performed with Student’s *t*-test. * *p* < 0.05 vs. VEGF-C-alone treated mice.

To test whether these kidney-targeting NPs could deliver the VEGFR-3-specific form of VEGF-C (VEGF-C c156s), and increase renal lymphatic density in hypertensive mice, we made male and female mice hypertensive with either angiotensin II via osmotic pump implanted subcutaneously (A2HTN) or the nitric oxide inhibitor L-NAME in the drinking water (LHTN). Nanoparticle-only control mice were omitted, as it has previously been reported that PLGA-PEG-FOL nanoparticles do not alter kidney morphology when injected intravenously on a similar injection schedule [21]. After systolic blood pressure was increased significantly in both groups (2 days), mice received either free VEGF-C protein alone or VEGF-C encapsulated in the NPs by tail vein injection every 3 days. On Day 16, after 4 injections, all mice were euthanized, and tissues collected. Podoplanin immunolabeling of lymphatic vessels in the renal cortex demonstrated increased lymphangiogenesis in hypertensive mice that received VEGF-C in NPs compared to mice that received free VEGF-C protein alone (Figure 2A). Quantitation of podoplanin-positive pixels in the renal cortex confirmed a significant increase (Figure 2B). Additionally, lymphatic vessels in the renal cortex of nanoparticle-treated mice had a significantly greater maximal lymphatic vessel width (Figure 2C). With increased renal VEGF-C, this is likely due to lymphatic vessel hyperplasia, but increased lymph volume could also play a role [12,22]. Together, these data support that the kidney-targeted NPs were able to promote lymphangiogenesis primarily in the kidney, as there was no evidence of increased lymphatic density in the heart, liver, lung, or skin (Figure S5).

**Figure 3:**
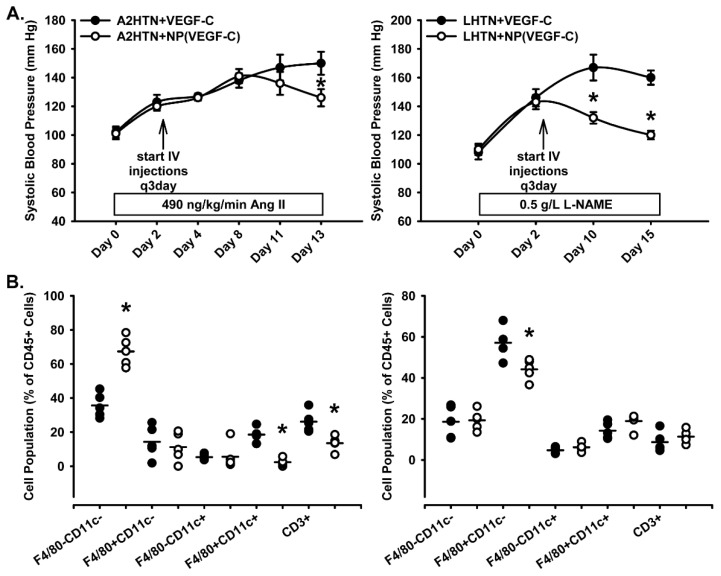
Following treatment with VEGF-C in NPs, significantly decreased blood pressure, and favorably altered renal immune cells, were observed. (**A**) Systolic blood pressure measures in male and female A2HTN and LHTN mice treated every 3 days with VEGF-C in NPs or VEGF-C alone. (**B**) Immune cell populations expressed as a percentage of CD45+ cells in kidneys of male and female A2HTN and LHTN mice treated every 3 days with VEGF-C in NPs or VEGF-C alone as determined by flow cytometry. Results are expressed as mean ± SEM or mean (n = 4–5 per group) and statistical analyses were performed with Student’s *t*-test or one-way ANOVA. * *p* < 0.05 vs. VEGF-C-alone treated mice.

Treatment with VEGF-C in NPs or free VEGF-C protein alone had no effects on body weight, right or left kidney weight, or spleen weight in hypertensive mice (Tables S2 and S3). We examined whether this NP-mediated increase in renal lymphatic density influenced blood pressure in both models of hypertension. Significantly decreased systolic blood pressure at day 13 was observed in male and female A2HTN mice treated with VEGF-C in NPs compared to male and female A2HTN mice treated with free VEGF-C protein alone (Figure 3A). In male and female mice with LHTN, significantly decreased systolic blood pressure by day 10 and persisted through day 15 was also observed following treatment with VEGF-C in NPs compared to male and female LHTN mice treated with free VEGF-C protein alone (Figure 3A).

Hypertension is associated with notable changes in renal immune cell populations. We previously reported that kidneys from mice with A2HTN exhibit a significant decrease in F4/80-CD11c- cells and a significant increase in F4/80+CD11c+ monocytes and CD3+ T cells compared to normotensive mice [9]. These effects were reversed by VEGF-C NP treatment, as kidneys from A2HTN mice had a significant increase in F4/80-CD11c- cells and a significant decrease in F4/80+CD11c+ monocytes and CD3+ T cells compared to A2HTN mice treated with free VEGF-C protein alone (Figure 3B). We also previously demonstrated that genetically inducing renal lymphangiogenesis in mice prevented the development of LHTN and this was associated with a significant decrease in renal F4/80+CD11c- monocytes [11]. In this study, kidneys from LHTN mice treated with VEGF-C in NPs also exhibited a significant decrease in these F4/80+CD11c- monocytes compared to LHTN treated with free VEGF-C protein alone (Figure 3B). These immune cell changes indicate increased efficiency of the lymphatic system within the NP-treated mice. F4/80+CD11c+ monocytes, F4/80+CD11c- monocytes, and most CD3+ T cells are traditionally pro-inflammatory immune cells and trafficking them out of the kidney may contribute to reduced inflammation [11]. These favorable improvements in renal immune cells may, in part, contribute to the decreased blood pressure in hypertensive mice.

**Figure 4:**
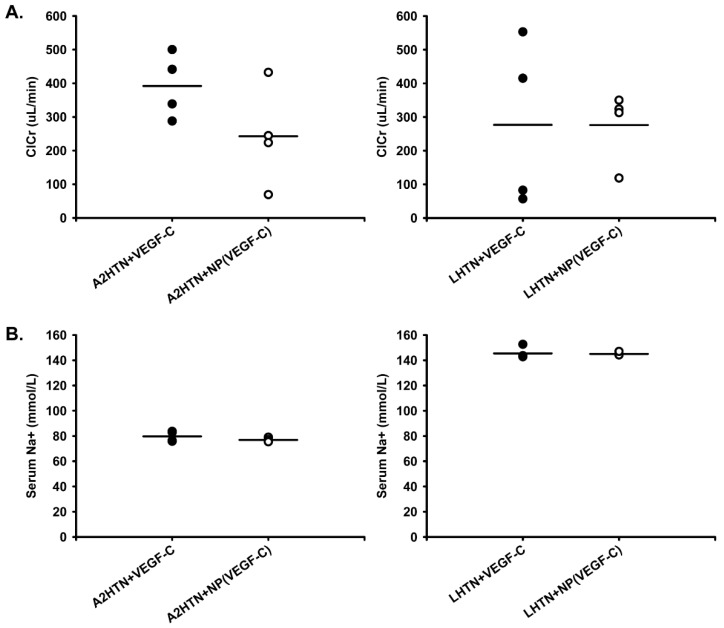
Treatment with VEGF-C in NPs had no effect on creatinine clearance or serum sodium levels. (**A**) Creatinine clearance and (**B**) serum sodium in male and female A2HTN and LHTN mice treated every 3 days with VEGF-C in NPs or VEGF-C alone. Results are expressed as mean (n = 4 per group) and statistical analyses were performed with Student’s *t*-test. * *p* < 0.05 vs. VEGF-C-alone treated mice.

**Figure 5:**
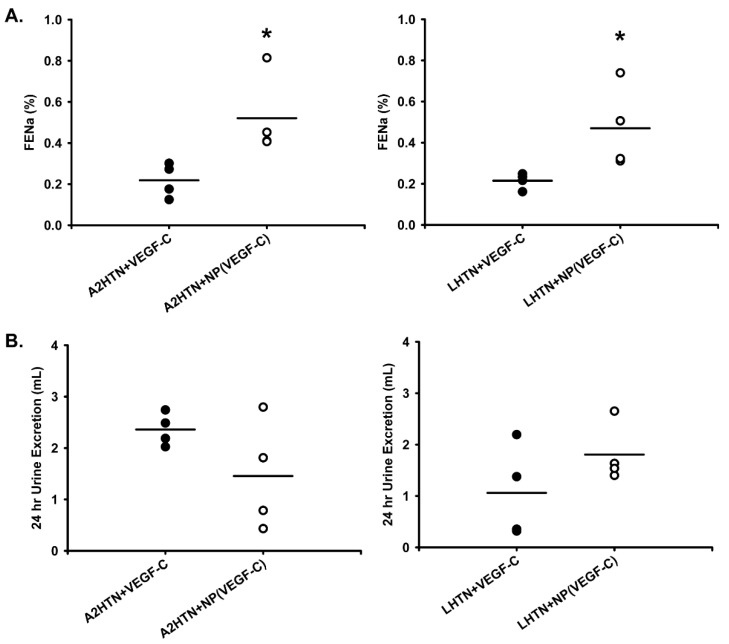
Treatment with VEGF-C in NPs significantly increased fractional excretion of sodium. (**A**) fractional excretion of sodium (FENa) and (**B**) 24 h urine excretion in male and female A2HTN and LHTN mice treated every 3 days with VEGF-C in NPs or VEGF-C alone. Results are expressed as mean (n = 4 per group), and statistical analyses were performed with Student’s *t*-test. * *p* < 0.05 vs. VEGF-C-alone treated mice.

Sodium balance is another important factor in blood pressure regulation with increased sodium retention playing a role in pathological hypertension. We reported previously that only under sodium-retaining conditions (i.e., LHTN and chronic salt loading) does genetically inducing renal lymphangiogenesis cause a significant doubling of fractional excretion of sodium (FENa) and a significant decrease in blood pressure [12]. In the current study, like our previous report, there was no difference in creatinine clearance (Figure 4A) or serum sodium (Figure 4B) between A2HTN and LHTN mice treated with VEGF-C in NPs or free VEGF-C protein alone. However, there was a significant increase in FENa in both A2HTN and LHTN mice treated with VEGF-C in NPs compared to those treated with free VEGF-C protein alone (Figure 5A). There were no significant differences in 24 h urine volume excreted between groups (Figure 5B). Consistent with an increased FENa in NP-treated mice, urinary sodium concentration in the 24 h collected sample was increased significantly in both A2HTN and LHTN mice treated with VEGF-C in NPs compared to those treated with free VEGF-C protein alone (Figure S6A). There were no significant differences between groups with respect to 24 h urinary sodium excretion, urinary creatinine, or serum creatinine (Figure S6B, S6C and S6D, respectively). These findings reinforce that, under hypertensive, sodium-retaining conditions, inducing renal lymphangiogenesis can benefit sodium handling and this may, in part, contribute to the anti-hypertensive effects.

## 4. Discussion

In this study, we demonstrated that the development and utilization of a NP that targets the kidney can deliver a pro-lymphangiogenic growth factor and cause an increase in renal lymphatic density. This therapy was able to significantly decrease blood pressure in mice with established hypertension, and this was associated with favorable changes in renal immune cells and increased fractional excretion of sodium. The development of NPs that primarily target the kidneys, in particular proximal tubule cells, has been reported previously [13].

Although sexual dimorphisms in the context of hypertension have been reported, males and females experience many of the same comorbidities associated with hypertension. Males and females have been combined previously in the case of LHTN, due to a lack of significant sex differences stemming from the disease pathology [11]. In a similar nitric oxide inhibition-induced hypertension model in rats, blood pressures between male and female groups were not significantly different until after Day 15 [23]. In a murine A2HTN model where angiotensin II was administered at a rate of 1.5 mg/kg/day, blood pressures were not significantly different throughout the 14-day study [24]. In past studies, we have observed compensatory renal lymphangiogenesis in both male and female LHTN and A2HTN mice, and renal lymphatic vessel density was not significantly altered between sexes [9,11]. Given this information, it was appropriate to combine males and females into equally divided groups to better represent the hypertensive population.

Therapies involving pro-lymphangiogenic VEGF-C have been reported to increase renal, skin, and cardiac lymphatics and lower blood pressure and/or reduce kidney injury. Beaini and colleagues reported that in salt-sensitive hypertensive mice, subcutaneous injections of recombinant VEGF-C every other day increased skin and renal lymphatics and this significantly decreased systolic blood pressure and renal injury [25]. Yang and colleagues reported that Spontaneously Hypertensive Rats (SHR) that received weekly intravenous injections of a VEGF-C retrovirus during the last 4 weeks of a 12-week high-salt diet had increased cardiac lymphatics and decreased systolic blood pressure from 197 to 189 mm Hg [26]. Hasegawa and colleagues delivered recombinant VEGF-C via an osmotic pump implanted into mice with unilateral ureteral obstruction, a model of renal fibrosis, and reported a significant reduction in renal interstitial fibrosis and inflammation compared to controls [27]. While these systemic delivery methods of VEGF-C were moderately effective, off-target effects were not explored fully, as VEGF-C has a short half-life and VEGFR-3 is expressed on several cell types in tissues throughout the body. The ability to specifically target the kidney for the delivery of a pro-lymphangiogenic factor may therefore represent a new anti-hypertensive therapeutic strategy.

Our previous work has described a theory linking increased lymphatics and renal sodium handling to blood pressure under sodium-retaining conditions [12]. In a diseased kidney, the interstitium becomes fibrotic and can expand upwards of 60%, depending on the severity of kidney damage [28]. An increase in fluid load during inflammatory conditions has been linked to an increase in lymphatic vessel diameter and consequently maximal lymphatic vessel width, which we observed in the current study [29]. Renal lymphangiogenesis has been associated with several chronic inflammatory diseases, including hypertension [11]. An augmented lymphatic network capable of transporting more lymph fluid could lead to an expanded interstitium. These changes in renal physiology would produce more renal tissue fluid, alter renal hydrodynamics, and require venous capillaries to reabsorb more fluid. Increased capillary reabsorption, in addition to increased lymphatic activity, would lead to an increase in venous return. To compensate for additional fluid in the system and the resulting myocardial stretching, the heart would release ANP. This ANP release could be the cause of the increased FENa and urinary sodium concentrations observed in the NP-treated mice of both hypertensive models.

A different theory explores the link between immune cell trafficking and blood pressure. It is well established that increased levels of activated renal immune cells are associated with inflammatory diseases, including hypertension. Removing and/or altering this immune response has been shown to prevent hypertension, thus confirming the criticality of activated renal immune cells to overall disease pathology [9,11,30]. The decrease in proinflammatory immune cells in the NP-treated mice may be a result of the enhanced renal lymphatic network in those mice. If the lymphatics are trafficking the pro-inflammatory immune cells out of the kidney efficiently, those cells would not have ample opportunity to cause inflammation via pro-inflammatory cytokine secretion; thus, blood pressure would decrease. Other theories regarding how renal-specific lymphangiogenesis can lower blood pressure include altered cytokine levels, altered RAAS levels, improved tubuloglomerular feedback, and the specific effects of VEGF-C on lymphatic pumping or other cell types.

VEGF-C is known to be correlated with tumorigenesis, though the specifics of this relationship are still being explored. Tumor interactions with VEGFR-2 and VEGFR-3 can vary depending on the organ of origin. Elevated levels of VEGFR-3 ligands have been associated with lymphangiogenesis in and around the primary tumor as well as in increasing tumor metastases through the lymphatic network [31]. Increased signaling is thus from the tumor to the lymphatic, with VEGFR-3 expression on cancer cells (of which there are few reports) having less relevance to progression [32]. While VEGF-C is known to induce intra-tumoral lymphangiogenesis, a VEGFR-3 specific form of VEGF-C has been shown not to induce tumor angiogenesis, which is crucial for metastasis [31]. Additionally, hypertension is known to increase the risk of renal cell carcinoma (RCC). Because hypertension is accompanied by an increase in renal lymphatic vessel density, the possibility of lymphatic involvement in RCC development cannot be ruled out. However, studies have shown that the role lymphangiogenesis plays in the progression of human RCC is minimal, and that VEGF-A (rather than B, C, or D) is associated with physiological characteristics of RCC [33]. Given this information, it is unlikely that delivery of VEGF-C156S would cause RCC; however, the long-term effects of treatment would need to be observed.

We have previously demonstrated that genetically inducing renal lymphangiogenesis can prevent experimental hypertension. However, until now, a kidney-specific, nanoparticle-based pharmacological therapy affecting lymphatics had not been developed to combat hypertension. Our nanoparticle, that targets the kidney, delivers a pro-lymphangiogenic growth factor, induces lymphangiogenesis, and attenuates hypertension, may be useful clinically. The nanoparticle materials are all approved by the United States Food and Drug Administration and the VEGF-C mutant is a recombinant protein, thus making the potential pharmaceutic scalable and inexpensive to mass produce. Of course, more preclinical and subsequent toxicology studies are necessary to move forward, but it has the potential to be an alternative or complementary anti-hypertensive therapy.

## Data Availability

The data presented in this study are available in FigShare at 10.6084/m9.figshare.16924300.

## Supplementary Materials

The following are available online at https://www.mdpi.com/article/10.3390/pharmaceutics14010084/s1, Figure S1: Certificate of analysis provided by PolySciTech; Figure S2: Schematic for nanoparticle assembly; Figure S3: Cumulative release of Coumarin 6 and FITC-ovalbumin from the folate nanoparticles; Figure S4: Gating strategy for renal immune cell analysis; Figure S5: No increase in extra-renal lymphatic vessel density in hypertensive mice treated with VEGF-C in nanoparticles; Figure S6: Treatment with VEGF-C in NPs significantly increased urinary sodium; Table S1: Flow cytometry panel description for mouse kidneys; Table S2: Body and tissue weights for the A2HTN group; Table S3: Body and tissue weights for the LHTN group.
