# Supplementary material for: A Kidney-Targeted Nanoparticle to Augment Renal Lymphatic Density Decreases Blood Pressure in Hypertensive Mice
Source: Pharmaceutics. 2021 Dec 30;14(1):84. doi: 10.3390/pharmaceutics14010084 (PMC8780399; doi:10.3390/pharmaceutics14010084)
Supplement: Supplementary file 1 [file pharmaceutics-14-00084-s001.zip › pharmaceutics-1472572-supplementary.pdf]

pharmaceutics

Supplementary Materials: A Kidney-Targeted Nanoparticle to Augment Renal Lymphatic Density Decreases Blood Pressure in Hypertensive Mice

Bethany L. Goodlett [1], Chang Sun Kang [2], Eunsoo Yoo [2], Shobana Navaneethabalakrishnan [1], Dakshnapriya Balasubbramanian [1], Sydney E. Love [1], Braden M. Sims [1], Daniela L. Avilez [1], Winter Tate [1], Delilah R. Chavez [1], Gaurav Baranwal [1], Mary B. Nabity [3], Joseph M. Rutkowski [1], Dongin Kim [2] and Brett M. Mitchell [1,*]

[1] Department of Medical Physiology, College of Medicine, Texas A&M University, Bryan, TX 77807, USA; bethany.goodlett@tamu.edu (B.L.G.); navshobi27@exchange.tamu.edu (S.N.); Dakshna.Bala@childrens.harvard.edu (D.B.); sydney.love99@tamu.edu (S.E.L.); bradensims@tamu.edu (B.M.S.); danielalaavilez@tamu.edu (D.L.A.); winterwinter97@tamu.edu (W.T.); delilahrchavez@tamu.edu (D.R.C.); gauravbaranwal@tamu.edu (G.B.); rutkowski@tamu.edu (J.M.R.); brettmitchell@tamu.edu (B.M.M.)

[2] Department of Pharmaceutical Sciences, College of Pharmacy, Texas A&M University, College Station, TX 77843, USA; chang-kang@ouhsc.edu (C.S.K.); eyoo@ncat.edu (E.Y.); Dongin-Kim@ouhsc.edu (D.K.)

[3] Department of Veterinary Pathobiology, College of Veterinary Medicine & Biomedical Science, Texas A&M University, College Station, TX 77843, USA; mnabity@cvm.tamu.edu (M.B.N.)

* Correspondence: Brett Mitchell, PhD, ORCID: 0000-0002-2575-8761, 8447 Riverside Parkway, Bryan, TX 77807, Ph:+ 1-979-436-0751, brettmitchell@tamu.edu

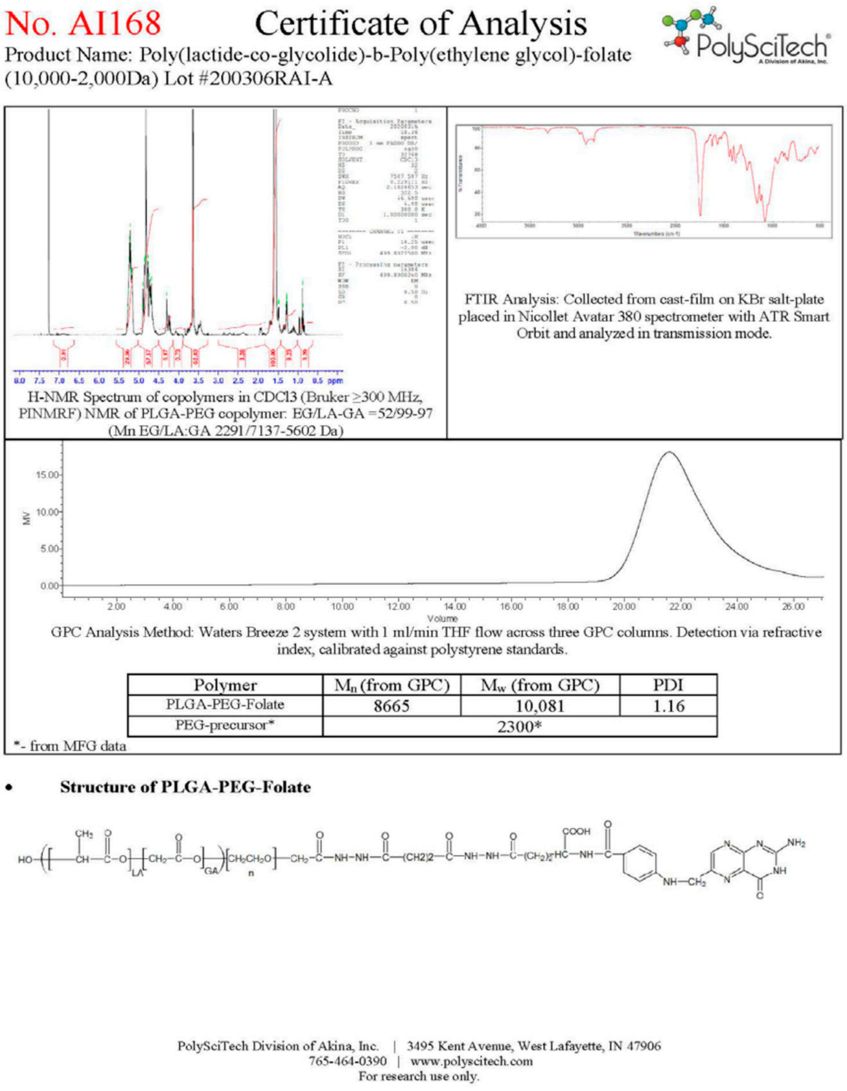

No. AI168 Certificate of Analysis

Product Name: Poly(lactide-co-glycolide)-b-Poly(ethylene glycol)-folate (10,000-2,000Da) Lot #200306RAI-A

H-NMR Spectrum of copolymers in CDCl3 (Bruker ≥300 MHz, PINMRF) NMR of PLGA-PEG copolymer: EG/LA-GA = 52/99-97 (Mn EG/LA:GA 2291/7137-5602 Da)

FTIR Analysis: Collected from cast-film on KBr salt-plate placed in Nicollet Avatar 380 spectrometer with ATR Smart Orbit and analyzed in transmission mode.

GPC Analysis Method: Waters Breeze 2 system with 1 ml/min THF flow across three GPC columns. Detection via refractive index, calibrated against polystyrene standards.

Polymer	M_n (from GPC)	M_w (from GPC)	PDI
PLGA-PEG-Folate	8665	10,081	1.16
PEG-precursor*		2300*	

*- from MFG data

- **Structure of PLGA-PEG-Folate**

PolySciTech Division of Akina, Inc. | 3495 Kent Avenue, West Lafayette, IN 47906
765-464-0390 | www.polyscitech.com
For research use only.

Figure S1. Certificate of analysis provided by PolySciTech. The NMR, FTIR analysis, and the GPC for the PLGA-PEG-folate polymer.

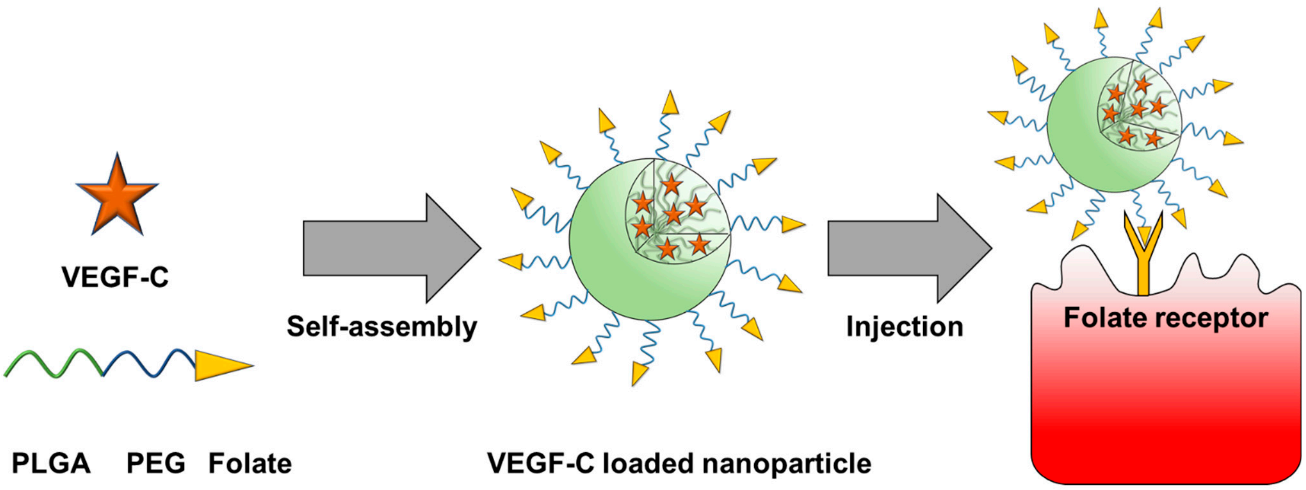

Figure S2. Schematic for nanoparticle assembly. PLGA, PEG, folate, and VEGF-C were utilized for the nanoparticle assembly followed by injection into the mouse.

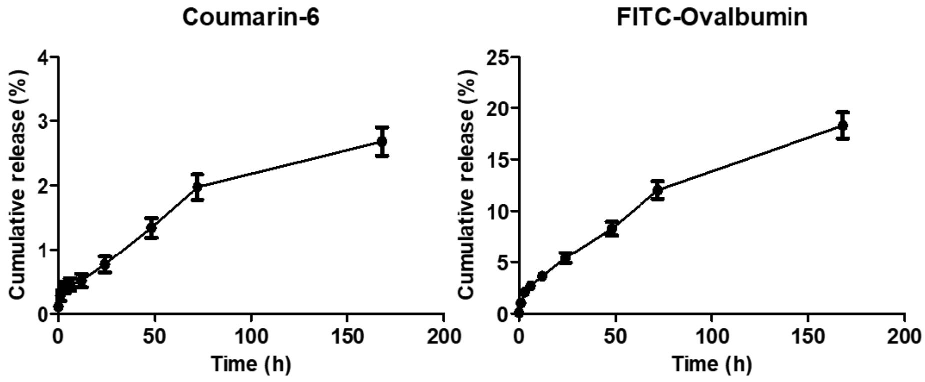

Figure S3. Cumulative release of Coumarin 6 and FITC-ovalbumin from the folate nanoparticles. The % of cumulative release over hours for both Coumarin 6 and FITC-ovalbumin.

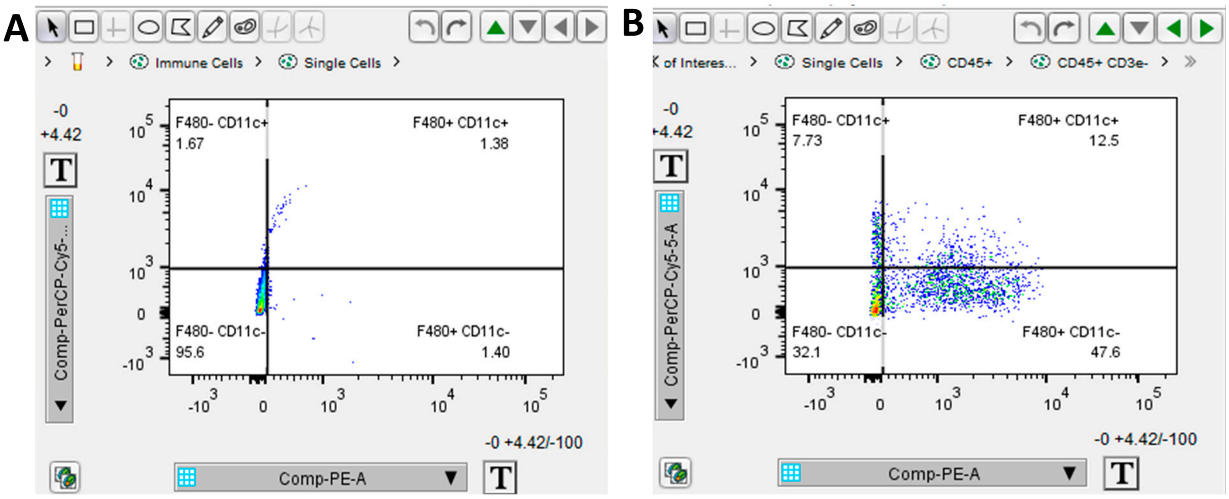

Figure S4. Gating strategy for renal immune cell analysis. (A) An unstained control kidney and (B) a fully stained kidney sample. Data was imported to FlowJo and gated for single, live cells. Then, a quadrant gate was used to determine the presence or absence of F4/80+ and CD11c+ cells. A similar approach was used for CD3+ T cells.

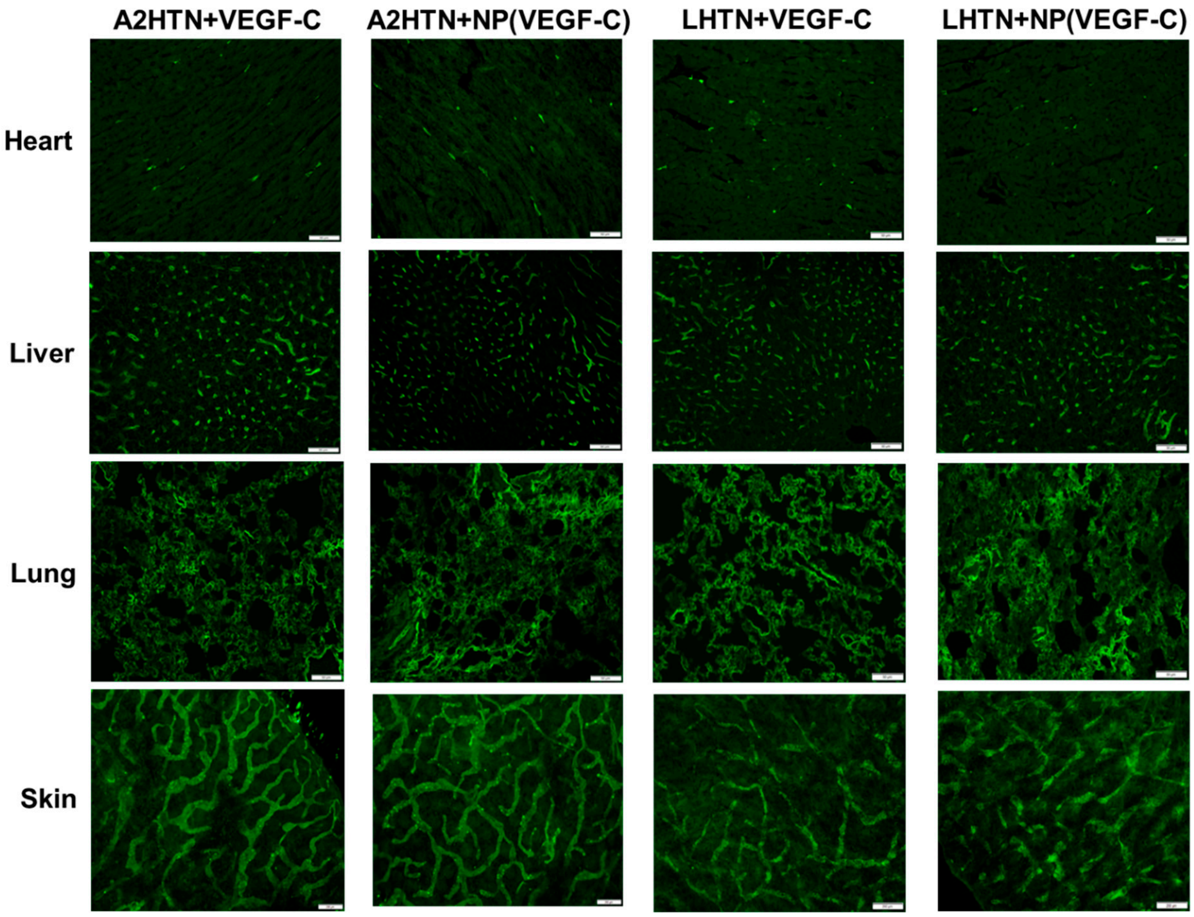

Figure S5. No increase in extra-renal lymphatic vessel density in hypertensive mice treated with VEGF-C in nanoparticles. LYVE-1 staining (green) in tissues from A2HTN and LHTN mice treated with VEGF-C in NPs or VEGF-C alone. Scale bars = 100 μm for heart, liver, and lung, and 200 μm for ear skin.

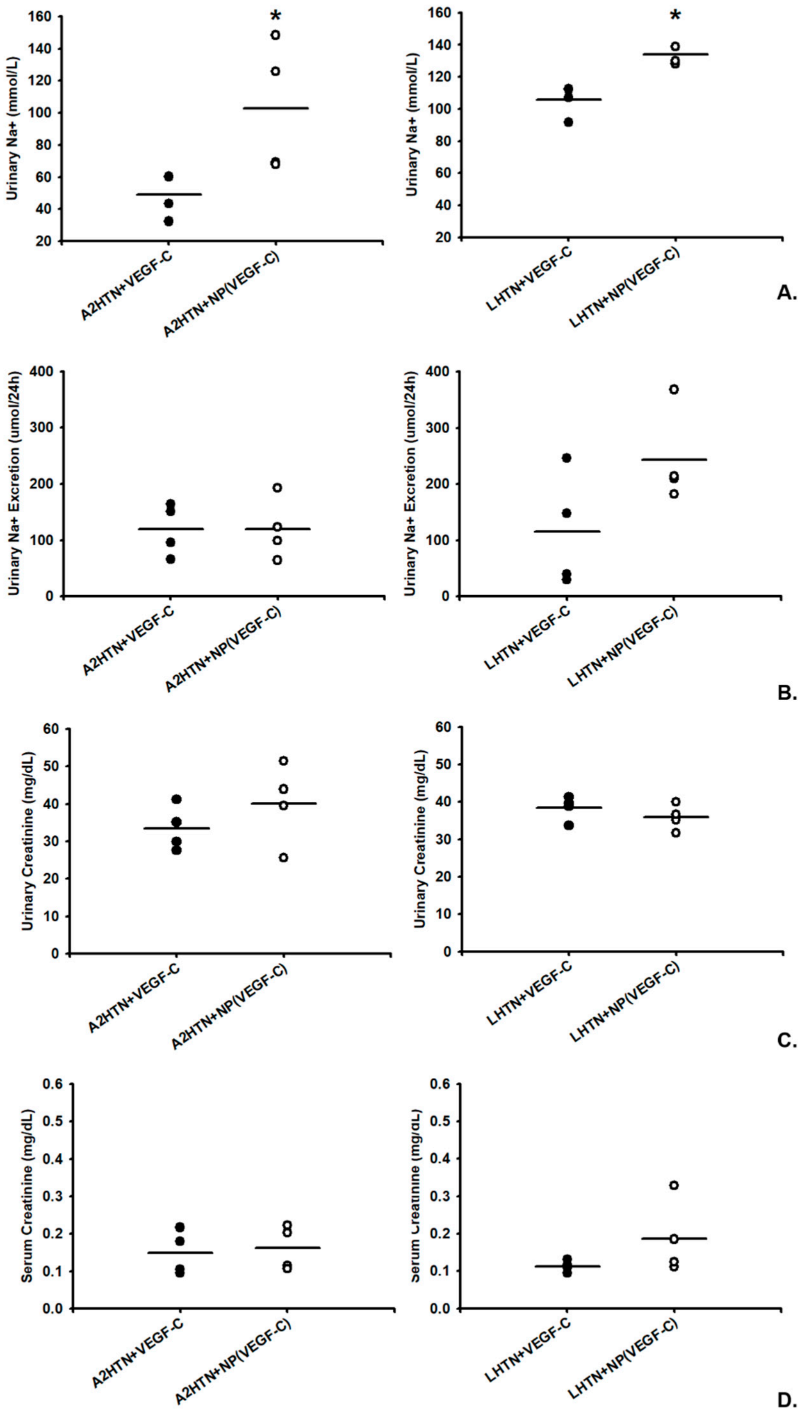

Figure S6. Treatment with VEGF-C in NPs significantly increased urinary sodium. (A) Urinary sodium, (B) urinary sodium excretion, (C) urinary creatinine, and (D) serum creatinine in male and female A2HTN and LHTN mice treated every 3 days with VEGF-C in NPs or VEGF-C alone. Urine samples were collected over a 24-hour period. Results are expressed as mean (n= 4-5 per group) and statistical analyses were performed with Student's *t* test. *P<0.05 vs VEGF-C alone treated mice.

Table S1. Flow cytometry panel description for mouse kidneys.

**Fluorochrome**	**FITC**	**PacBlue**	**PE**	**PerCP-Cy5.5**
**Antigen**	CD45.2	CD3e	F4/80	CD11c
**Final Conc (µg/mL)**	2.5	0.1	0.25	0.5
**Dilution Factor**	1:200	1:2,000	1:400	1:400
**Clone**	104	500A2	T45-2342	HL3

Abbreviations: FITC = fluorescein isothiocyanate; PacBlue = pacific blue; PE = phycoerythrin; PerCP-Cy5.5 = peridinin chlorophyll protein complex cyanine 5.5.

Table S2. Body and tissue weights for the A2HTN group.

	**Body Weight (BW) (g)**		**R Kidney (mg)/BW**		**L Kidney (mg)/BW**		**Spleen (mg)/BW**	
	**M**	**SE**	**M**	**SE**	**M**	**SE**	**M**	**SE**
A2HTN+VEGF-C	28.5	1.9	6.3	0.4	5.9	0.4	3.2	0.5
A2HTN+NP(VEGF-C)	27.1	3.1	6.1	0.4	5.4	0.4	3.6	0.4

Table S3. Body and tissue weights for the LHTN group.

	**Body Weight (BW) (g)**		**R Kidney (mg)/BW**		**L Kidney (mg)/BW**		**Spleen (mg)/BW**	
	**M**	**SE**	**M**	**SE**	**M**	**SE**	**M**	**SE**
LHTN+VEGF-C	27.1	2.5	6.7	0.3	7.1	0.2	3.4	0.5
LHTN+NP(VEGF-C)	27.5	3.5	6.3	0.2	7.4	0.4	2.6	0.5
